# Nasal septum perforation: a side effect of bevacizumab chemotherapy in breast cancer patients

**DOI:** 10.1038/sj.bjc.6605828

**Published:** 2010-08-24

**Authors:** A Mailliez, C Baldini, J T Van, V Servent, Y Mallet, J Bonneterre

**Affiliations:** 1 Breast Cancer Department, Centre Oscar Lambret, 3 rue F Combemale, Lille 59000, France; 2 Head and Neck Department, Centre Oscar Lambret, 3 rue F Combemale, Lille 59000, France; 3 Université de Lille Nord de France, Lille 59000, France

**Keywords:** nasal septum perforation, breast cancer, bevacizumab, antiangiogenic therapy toxicity, epistaxis

## Abstract

**Background:**

Bevacizumab is an anti-vascular endothelial growth factor approved in association with paclitaxel or docetaxel as first line in patients (pts) with metastatic breast cancer. Rare cases of nasal septum perforations have been reported. We report our experience of nasal perforation in breast cancer pts receiving bevacizumab and chemotherapy either in the adjuvant or in the metastatic settings.

**Methods:**

Between 1 January and 31 December 2009, 70 pts received bevacizumab together with chemotherapy. All the pts who had received bevacizumab were referred to the ENT specialist. Symptoms potentially related were looked for. Side effects were graded according to CTCAE.

**Results:**

Five nasal septum perforations were diagnosed (5 out of 70; 7.14%). Bevacizumab dose was 15 mg kg^−1^ 3 weekly. Three pts were metastatic. Bevacizumab was associated with docetaxel (100 mg m^−2^ every 3 weeks) in two pts and with weekly paclitaxel in one. The last two pts received bevacizumab in combination with anthracyclin and then taxanes in the adjuvant setting. In these two cases, nasal septum perforation occurred at the time of docetaxel treatment.

**Conclusion:**

A high incidence of nasal septum perforation has been shown in pts with breast cancer receiving bevacizumab together with chemotherapy. Several mechanisms could be involved (mucositis, delayed tissue repair, antiangiogenic action of taxanes).

Angiogenesis is a hallmark of cancer. Among the angiogenesis factors identified, vascular endothelial growth factor (VEGF) is a crucial regulator of angiogenesis in normal and malignant tissues (Plate *et al*, 1992; Ferrara and Alitalo, 1999). Bevacizumab is a humanised monoclonal antibody directed against VEGF, which has significant activity against several solid tumours. Bevacizumab in association with paclitaxel or docetaxel has been approved as first line in patients (pts) with metastatic breast cancer. A significant benefit has been observed on relapse-free survival ((RFS) when combined with paclitaxel and docetaxel; Miller *et al*, 2007; Miles *et al*, 2009). Although not approved yet, bevacizumab has been shown to give a statistically significant benefit on RFS when given as a second line together with anthracyclines or capecitabine (Brufsky *et al*, 2009). It is currently used in clinical studies in the adjuvant or neoadjuvant setting. Observed toxicities of bevacizumab in the first studies include hypertension, proteinuria, mild to moderate bleeding, delayed wound healing and thromboembolic events. Rare cases of nasal septum perforations have been reported (Fakih and Lombardo, 2006; Traina *et al*, 2006; Ruiz *et al*, 2007; Burkart *et al*, 2008; Marín *et al*, 2009; Power and Kemeny, 2010). We report our experience of nasal perforation in breast cancer pts receiving bevacizumab and chemotherapy either in the adjuvant or metastatic settings.

## Patients and methods

### Patients

Between 1 January and 31 December 2009, 70 pts received bevacizumab every 3 weeks together with chemotherapy, 67 at a dose of 15 mg kg^−1^ and 3 at 7.5 mg kg^−1^. In all, 15 pts had bevacizumab alone after the end of chemotherapy. After the observation of the first nasal perforation cases, the clinical files of all the pts who had received bevacizumab were reviewed. All of the pts treated or having been treated with Bevacizumab were referred to the ENT specialist for a nasal examination. Symptoms potentially related like nasal obstruction, rhinorrhea, mucosal crusting and epistaxis were looked for. The clinical characteristics of the pts and the details of the treatments are given in Table 1.

### Methods

The first administration of bevacizumab was a 90 min intravenous infusion, which was followed by the chemotherapy regimen. The second administration of bevacizumab lasted 60 min and the duration of the following ones was 30 min. Bevacizumab dose was 15 mg kg^−1^ except for three pts in a clinical study who received 7.5 mg kg^−1^. Docetaxel was given at a dose of 100 mg m^−2^ every 3 weeks during 2 h, weekly Paclitaxel (90 mg m^−2^) administration lasted 1 h. Capecitabine was given at a dose of 1500 mg twice daily, 2 weeks out of 3.

Side effects were graded according to CTCAE (Cancer Therapy Evaluation Program, 2006).

Before each course, leukopenia, proteinuria and hypertension were monitored and the worst grade observed during the courses was considered, as well as other side effects.

### Results

All 70 pts received bevacizumab for breast cancer. Median age was 54 years (range, 32–74 years). All pts were female. One pt. had a history of chronic sinusitis before bevacizumab/chemotherapy. In all, 52 (75%) were treated in the metastatic setting (12 first line, 27 second line and 13 had received more than two lines). In all, 12 pts were included in an adjuvant clinical study. Six received bevacizumab with a neoadjuvant chemotherapy in a clinical study too. In all, 15 pts received weekly paclitaxel, 36 docetaxel every 21 days, 1 capecitabine. All pts in clinical studies received bevacizumab in combination with anthracyclines and then in association with docetaxel.

### Clinical characteristics of pts with nasal septum perforation

**Figure 1:**
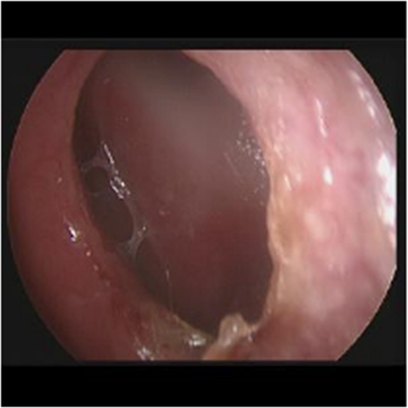
Nasal septum perforation.

Five nasal septum perforations were found in the 70 pts (7.14% Figure 1). At diagnosis, Bevacizumab was part of the treatment of the five pts. Bevacizumab dose was 15 mg kg^−1^ 3 weekly in all the pts. The median time between beginning of Bevacizumab treatment and diagnosis of nasal septum perforation was 21 weeks (range, 9–45 weeks). The median age of affected pts was 42 years (range, 33–50 years). Three pts were metastatic and had received previous chemotherapy in the adjuvant setting (anthracyclin based for one and sequential anthracyclin then docetaxel for the others). One of them had epistaxis and two of them developed mucosal toxicities (grade1 or 2) in the adjuvant setting. Bevacizumab was associated with docetaxel (100 mg m^−2^ every 3 weeks) in two pts and with weekly paclitaxel for one pt.

The last two pts received bevacizumab in combination with anthracyclin and then taxanes in the adjuvant setting. In these two cases, nasal septum perforation occurred at the time of docetaxel association.

None of the pts developed hypertension or proteinuria. Related toxicities were mucositis (stomatitis four pts, one grade2 and three grade1), cutaneous (four pts, one grade1 and three grade2), haematological (two neutropenia, one grade3 and one grade4) and neurological (one dysesthesia grade2). All but one had epistaxis. One pt. described disorders of nasal airflow and wheezing. One had crusting and infection of nasal mucosa. The total dose received at the time of nasal perforation diagnosis was 45, 105, 120, 150 and 225 mg kg^−1^, respectively.

Bevacizumab was stopped in four pts after the diagnosis of nasal perforation. In one pt, bevacizumab was continued because of a very good clinical response. Patients with nasal septum perforation were followed by the ENT specialist; up to now, no healing and no extension was observed even in the pt who continued bevacizumab treatment.

### Clinical characteristics of pts without nasal perforation

Among the other pts who received bevacizumab and chemotherapy without nasal septum perforation, 15 pts died, all of progressive disease. Nine of them had epistaxis. Only two had ENT examination and no nasal septum perforation was diagnosed.

**Figure 2:**
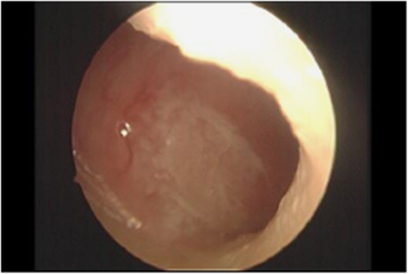
Nasal septum irritation.

Among the 50 alive pts without nasal septum perforation, 41 pts (82%) had epistaxis, 11 nasal crusting (Figure 2). Among the 27 pts who had received previous chemotherapy, only 1 experienced epistaxis (both with docetaxel and FEC) and 4 had mucosal toxicity. In all, 48 had ENT examination.

A total of 30 pts out of 50 (60%) had mucosal toxicity (13 grade1, 8 grade2 and 9 grade3). In all, 16 experienced cutaneous toxicities (2 grade1, 8 grade2 and 6 grade3), 8 haematological toxicities (1 neutropenia grade3 and 7 grade4), 1 neurological toxicity (neuropathy grade3) and 1 cardiac event (pericarditis). Three pts had proteinuria (max 1.5 g per 24 h). Hypertension appeared at least at one time for 15 pts (grade1–2; TA max 196/96; 30%).

### Response to treatment

Response to treatment could be assessed in pts with metastatic disease and in pts in the neoadjuvant setting. The response rates were in first-line metastatic 7 partial responses (PRs); in second line 2 complete responses (CRs), 15 PRs, 1 stable disease and 1 progression; after the second line, 2 CRs, 5 PRs and 1 progression. In all, two CRs and four PRs were observed in pts receiving a neoadjuvant treatment.

## Discussion

This study reports our experience of nasal septum perforations in breast cancer pts receiving bevacizumab and chemotherapy. The incidence rate of nasal septum perforation was 7.14%. To our knowledge, such a high rate has never been reported and might be underestimated.

To our knowledge, only eight cases of nasal septum perforation in bevacizumab-treated pts have been reported. The clinical characteristics are summarised in Table 2. There are two cases of breast cancer, five cases of colon cancer and one case of ovarian cancer.

Nasal septum perforations with bevacizumab can thus occur independently of the chemotherapy regimen (LV5FU2, FOLFOX, paclitaxel) and sometimes during maintenance therapy with bevacizumab. The total dose exposure before perforation varies from 30 to 150 mg kg^−1^.

The management is not clearly defined. In some cases bevacizumab was discontinued. We do not know whether bevacizumab must be stopped or which treatment should be proposed.

ENT monitoring and evolution of the perforation (size, long term symptoms,…) are not detailed. In one case, plastic surgery was performed.

Nasal septum consists of an osteochondral skeleton covered with periosteum, perichondrum and mucosa. Its vascularisation depends on several arteries (Legent *et al*, 1999; Steff, 2000–2001): the sphenopalatine artery, a branch of the external carotid artery, the anterior and posterior ethmoidal artery, branches of the internal carotid artery. The anterior part of the nasal septum is highly vascularised with arterial anastomoses. Risk factors for nasal septum perforations are traumatic injury, intranasal drug use, inhaled irritants, inflammatory diseases and infections. Cocaine and other similar drugs are potent vasoconstrictors, which if applied on the mucosa of the nasal septum may cause intense inflammation, congestion and bacterial colonisation (Lorenzi *et al*, 1997). This can lead to ischaemic necrosis of the septal cartilage. Inhalation of chemical irritants (chromium, dust and metal oxide.) causes irritation and severe inflammation of the nasal mucosa responsible for ulcerations, which can lead to perforation of the nasal septum.

Nasal septum infection weakens the nasal mucosa and then can be responsible for perforation. Various systemic diseases can also cause nasal septal perforation (Wegener's granulomatosis, systemic lupus erythematosus, antiphospholipid syndrom, sarcoidosis… Vignes *et al*, 2002). Mechanisms involved are ischaemic, infections and inflammatory.

Younger and Blokmanis (2005) also noted that 3% of NSP may be completely asymptomatic and discovered during a consultation for another reason.

In our pts receiving antiangiogenic therapy and chemotherapy, several specific mechanisms could be involved. First, mucositis induced by chemotherapy may weaken nasal mucosa. Nasal irritation due to chemotherapy induced mucositis and/or bevacizumab may induce mucosal breaks and ulcerations. These irritations can be increased by frequent nose blowing and mechanical trauma. Neutropenia frequently associated with chemotherapy enhance the risk of local infection. Bevacizumab decreases normal tissue repair. Bleeding could be seen because of the effect of bevacizumab in wound healing. The inhibition of VEGF can induce a loss of capillaries by apoptosis of endothelial cells, which causes tissue necrosis. Cartilage is not vascularised. The trophicity is maintained by factors from capillaries of mucosa via a terminal vascularisation. In the weakened mucosa, the trophicity of the cartilage might no longer be obtained.

Finally, in our study, bevacizumab was associated with taxanes in most cases. Several studies have shown the antiangiogenic action of taxanes (Belotti *et al*, 1996; Hotchkiss *et al*, 2002; Grant *et al*, 2003). Docetaxel appears to be more potent than Paclitaxel (Grant *et al*, 2003). Endothelial cells seem to be 10–100-fold more sensitive to taxanes than tumour cells (Grant *et al*, 2003). Furthermore, two cases of nasal septum perforations have already been described with Docetaxel alone in pts without risk factors (Tan *et al*, 2006). A case of thrombotic microangiopathy following docetaxel has been reported (Siau and Varughese, 2009). This endothelial toxicity of taxanes might explain potentialisation of bevacizumab by taxanes in terms of efficiency, but also of toxicity including nasal septum perforation. To our knowledge, no other cases of nasal septum perforation with chemotherapy alone have been reported.

A high incidence of nasal septum perforation has been shown in pts with breast cancer receiving bevacizumab. Many questions remain about the prevention and the management of nasal septum perforation. A collaboration with ENT specialist is needed. Whether or not bevacizumab should be stopped remains a matter of debate. In the clinical practise, indication of antiangiogenic therapy in breast cancer is increasing and many pts are concerned, and clinicians must be aware of this side effect. ENT examinations must be systematically performed in all pts receiving bevacizumab, at least together with taxanes.

## Figures and Tables

**Table 1:** Clinical characteristics and details of treatments (70 patients)

Median age (min–max)	54 years (32–74)
*Treatment setting*	
Neoadjuvant	6
Adjuvant	12
Metastatic	52
	
*Number of previous lines of chemotherapy*	
0	12
1	27
⩾2	13
	
*Regimen*	
*Sequential anthracyclin/docetaxel* + *concomitant Bevacizumab*	
Neo adjuvant treatment	6
Adjuvant treatment	12
Docetaxel/Bevacizumab	37
Paclitaxel/Bevacizumab	14
Capecitabine/Bevacizumab	1
Bevacizumab as maintenance treatment	14

**Table 2:** Previous cases reported

	**Cases**	**Regimen**	**Symptoms**	**Management**
Fakih and Lombardo (2006)	Man, 53 years, metastatic colon cancer	FOLFOX + Bevacizumab	Nasal mucositis, occasional epistaxis	Not discussed
Traina *et al* (2006)	Women, 54 years, metastatic breast cancer	Paclitaxel + Bevacizumab	Rhinorrhea, nasal irritation, occasional epistaxis, alopecia of the nasal passage	Discontinuation of Bevacizumab + local supportive therapy
Ruiz *et al* (2007)	Man, 53 years, metastatic colon cancer	LV5FU2 + Bevacizumab	Occasional epistaxis + nose pain + fungal infection	Discontinuation of Bevacizumab + surgical excision of macroscopic pathological tissue + antifungal treatment
Burkart *et al* (2008)	Woman, 52 years, metastatic ovarian cancer	Bevacizumab	Epistaxis	Local supportive therapy + observation
Marín *et al* (2009)	Woman, 39 years, metastatic breast cancer	Paclitaxel + Bevacizumab	None	No treatment Bevacizumab discontinued for progression disease
Power and Kemeny (2010)	Woman, 68 years, metastatic colon cancer	FOLFOX + Bevacizumab	Running nose, intermittent epistaxis	Local supportive therapy + discontinuation of Bevacizumab and resuming at progressive disease
	Man, 40 years, metastatic colon cancer	LV5FU2 + Bevacizumab	Nasal pain, rhinorrhea, intermittent epistaxis	Local supportive therapy
	Man, 35 years, metastatic colon cancer	FOLFOX + Bevacizumab then FOLFIRI + Bevacizumab	Intermittent epistaxis, nasal congestion, crusting, whistling sound on nasal inspiration	Local supportive therapy Bevacizumab discontinued due to pulmonary embolus
